# Adherence to oral second-generation antipsychotic medications in patients with schizophrenia and bipolar disorder: physicians’ perceptions of adherence vs. pharmacy claims

**DOI:** 10.1111/j.1742-1241.2012.02918.x

**Published:** 2012-06

**Authors:** J J Stephenson, O Tuncelli, T Gu, D Eisenberg, J Panish, C Crivera, R Dirani

**Affiliations:** 1 HealthCore, Inc.Wilmington, DE, USA; 2 Janssen Scientific Affairs, LLCTitusville, NJ, USA; 3 Janssen Scientific Affairs, LLCRaritan, NJ, USA

## Abstract

**Objective:**

To compare physician-reported adherence of specific patients to oral second-generation antipsychotics vs. actual adherence rates determined from the patients’ pharmacy claims.

**Methods:**

Claims data from the HealthCore Integrated Research Database identified patients with schizophrenia or bipolar disorder with ≥1 oral second-generation antipsychotic prescription. The prescribing physicians were identified from the pharmacy claims and asked to complete an Internet survey assessing their perception of medication adherence for 1–2 of their patients and their beliefs regarding adherence to second-generation antipsychotics in general for a 1-year period. Adherence to second-generation antipsychotics was determined for each patient by pharmacy claims for the same period. Physician survey data were merged with patient claims data via unique patient identifiers, and physician-reported adherence rates were compared with claims-based rates as measured by the medication possession ratio.

**Results:**

One hundred and fifty-three physicians responded to the survey, representing 214 patients (44 with claims for schizophrenia, 162 with bipolar disorder, 8 with claims for bipolar disorder and schizophrenia). Most physicians (60%) had no formal adherence training. More than two-thirds (68%) reported emphasising the importance of adherence and reported approximately 76% of their patients were adherent (≥71% of the time). In the schizophrenia group, 16 of 17 (94%) patients with low-to-moderate (≤70%) adherence levels had high (≥71%) physician-estimated adherence. In the bipolar disorder group, 62 of 92 (67%) patients with low-to-moderate adherence levels had high physician-estimated adherence.

**Conclusions/Interpretation:**

These analyses suggest that, even when physicians are asked about specific patients in their practice, there is discordance between physician perceptions and adherence as measured through pharmacy claims. This disparity may delay appropriate interventions, potentially contributing to relapses.

**What’s known**

Medication adherence is particularly challenging in patients with schizophrenia or bipolar disorder, with as many as 50% of patients failing to take their medications as prescribed. Physicians typically overestimate patient adherence, even when assessing general adherence rates in their practises. Failure to recognise the role of non-adherence in symptom relapse may prompt physicians to misattribute poor outcomes to treatment failure, leading to inappropriate dosage increases or unnecessary medication switches.

**What’s new**

In a novel approach, physicians’ estimates of adherence rates for specific patients were compared with pharmacy claims for those same patients. Despite having an established therapeutic relationship with each patient, physicians still overestimated treatment adherence. Neither physician experience nor formal training in maximising patient adherence improved the ability of physicians to estimate adherence rates.

## Introduction

Mental health disorders account for a substantial portion of all health care expenditures in the United States (1). Although patients with schizophrenia and bipolar disorder make up only a fraction of the total US adult population, these patients utilise a large proportion of mental health resources (2, 3). Seeking health services is an important step in treating mental health conditions, but it is only part of an effective therapeutic regimen. Patients must actively take medications as prescribed, which includes not only following the prescribed dosing schedule but also taking the full dose and refilling prescriptions so that no treatment gaps occur (4). Adherence is especially challenging in patients with schizophrenia or bipolar disorder.

The costs of non-adherence are high (4). Non-adherence is associated with increased symptom recurrence, a higher risk of relapse and a greater likelihood of emergency room use and hospital admissions (5–8). Most patients with schizophrenia who fail to adhere to therapy suffer from relapse severe enough to require hospitalisation (9). Use of the second-generation antipsychotics, which have a different adverse event profile than the first-generation antipsychotics, was hoped to improve adherence (10) and, consequently, treatment outcomes compared with first-generation antipsychotics (6, 11). However, treatment adherence remains low (6). After 12 months of therapy, adherence rates with second-generation antipsychotics hover between 50% (12, 13) and 60% (14, 15). The adherence rates for antidepressants are slightly higher, approximately 65% (16).

Numerous studies have attempted to define reasons for non-adherence and to outline strategies for improving adherence rates (11, 14, 17). Such strategies are of limited use, however, if physicians are unable to accurately determine whether their own patients are adhering to treatment. Physicians overestimate patient adherence to therapy in general (4, 18–21), which becomes a problem when treatment decisions are based on therapeutic effectiveness, particularly in patients with schizophrenia or schizoaffective disorder. In a recent study, Byerly and colleagues found prescribers vastly overestimated patient adherence to antipsychotic therapy compared with electronically monitored pill bottle caps (22). Physician estimates of adherence were comparable to patient self-reports. Another study comparing physician estimates of adherence with electronic monitoring concluded that overall, physicians identified a similar proportion of adherent patients as electronic monitoring. However, when the study population was divided according to adherent and non-adherent patients based on pill counts, physicians failed to identify non-adherent patients. Thus, although the overall proportions were similar, physicians and electronic records identified different patients as being non-adherent (23). By not recognising the role of non-adherence in poor treatment outcomes, physicians may switch a patient’s medication or alter the dosage when the lack of effect is attributable not to treatment failure but rather to the patient’s failure to adhere to the regimen (20, 21).

If physicians tend to overestimate adherence in their wider practice, the question then becomes how well physicians estimate adherence for specific patients. Previous studies found physicians overestimated adherence among their patients with schizophrenia compared with objective measures such as pill counts or electronic monitors (22, 23). However, the assessment periods were short. After seeing a patient regularly for an extended period, physicians should be able to use their knowledge of that patient’s personality, symptoms, general demeanour during office visits and course of their condition to formulate an impression about how well that individual adheres to the prescribed medication regimen. Assessing the congruence between physicians’ perceptions of the adherence rates of specific patients and actual adherence rates as determined from the patients’ pharmacy claims data may be useful to determine what, if any, interventions are needed to improve adherence. Our study used a novel approach by determining the level of congruence between a physician’s estimate of adherence for individual patients and each patient’s pharmacy claims for the same period. The primary goal was to assess the accuracy of physicians’ perceptions of medication adherence for specific individuals with schizophrenia or bipolar disorder who were seen on a regular basis vs. patients in general and to compare the physicians’ perceptions of individual adherence rates with the corresponding pharmacy claims data.

## Methods

### Data source

This study utilised administrative claims data from the HealthCore Integrated Research Database (HIRD) for services incurred between May 1, 2008, and April 30, 2009, to identify commercially insured patients with schizophrenia or bipolar disorder between the ages of 18 and 64 years who used oral second-generation antipsychotics and the physicians who prescribed the medications. The broad, fully integrated HIRD consists of the eligibility, medical and pharmacy claims of approximately 30 million patients from 14 geographically dispersed health plans in the Northeastern, Midwestern, Southern and Western regions of the United States.

### Patient and physician identification

Eligible patients had to have at least two medical claims with different dates of service with an *International Classification of Diseases, 9th Revision, Clinical Modification* (ICD-9-CM) diagnostic code for schizophrenia (ICD-9-CM 295.xx) or bipolar disorder (ICD-9-CM 296.0, 296.1x or 296.9x), or both, and at least two pharmacy claims for an oral second-generation antipsychotic (i.e., olanzapine, risperidone, quetiapine, ziprasidone, aripiprazole or paliperidone) between May 1, 2008, and April 30, 2009. The latest pharmacy claim was defined as the index pharmacy claim, and its date the index date. All patients were between 18 and 64 years of age as of the index date and were covered by employer-provided insurance and had at least 12 months of continuous health plan eligibility before the index date, with pharmacy claims data for at least 6 months before the index date. Patients who had received a long-acting injectable antipsychotic during the study period were excluded.

The physician sample list was determined from prescribing physician information on the index pharmacy claims of eligible patients. Physicians with at least two patients who fulfilled the above inclusion and exclusion criteria were identified as potential physician survey participants.

### Physician survey methodology

Physicians were ranked in descending order according to the number of eligible patients with claims for schizophrenia and bipolar disorder. All eligible physicians had at least two patients with ICD-9-CM codes for schizophrenia or bipolar disorder, or both. If a physician had more than five patients meeting the study inclusion and exclusion criteria, then five patients were chosen at random from that physician’s patient list for use in the physician survey.

Physicians were faxed an invitation, including a URL, to participate in the online survey. Each physician received a unique identifier and password to prevent multiple survey submissions. Physicians who visited the URL provided consent to participate via an online checkbox and completed the approximately 25-min survey for a maximum of two patients. The first three questions were screens to ensure that the identified patient was a current patient of this physician, had been this physician’s patient for at least the past 12 months and had the specified diagnosis. If the physician responded ‘yes’ to all three screening questions, the physician was then asked a series of questions about that patient’s adherence to oral atypical antipsychotics. If the physician responded ‘no’ to any of the three screening questions, the physician was returned to the start of the screening questions and, if applicable, another patient’s name appeared or the survey was terminated.

The physician survey assessed physician perceptions of the level of medication adherence for a maximum of two specific patients in their practice and the physician’s beliefs, attitudes and behaviours regarding medication adherence in general. The patient’s name appeared only on the first screening question. After that question, the patient was referred to as Patient A or Patient B to limit the visibility of the patient’s actual name. Physicians did not receive any indication of their patients’ actual medication adherence as determined from the patients’ pharmacy claims.

### Institutional Review Board Approval

Because patients’ personal health information was required for the conduct of this study, a Health Insurance Portability and Accountability Act (HIPAA) Waiver of Authorization was received from a central institutional review board before accessing any personal health information.

### Outcome measures

The physician’s perception of adherence was determined by the question, ‘During the past 12 months, what percentage of the time do you think [Patient A/Patient B] has been adherent to his/her oral second-generation antipsychotic therapy?’ The physician endorsed the response category that most closely corresponded to their perception of the adherence of [Patient A/Patient B] where the response categories were presented in deciles that ranged from a low of ‘0–10% of the time’ to a high of ‘91–100% of the time’.

Actual oral second-generation antipsychotic use was determined from the patient’s pharmacy claims by the number of prescription fills, number of days of medication supply and the total dose units for the same 12-month period. Claims-based adherence was determined using the medication possession ratio (MPR). The MPR was calculated by dividing the number of days covered (the number of days the patient had a supply of the index medication during the 12-month period prior to the date of the survey) by 365 (24). MPR scores ranged from 0% to 100% and were categorised by deciles to correspond to the physician adherence categories. Categories were further summarised into low, medium and high adherence where low adherence was defined as ranging from 0–30%, moderate adherence from 31–70% and high adherence from 71–100%. Medication discontinuation was defined as any gap in antipsychotic claims longer than the allowable interval based on the prescribed supply.

Physician survey data for specific patients were merged with the patient-specific administrative claims data via a patient identifier to evaluate the level of congruence between the physician’s perception of adherence and the patient’s pharmacy claims-based adherence, as measured by the MPR. Physicians were grouped according to patient levels of adherence. Physician-patient pairs were then classified according to the concordance of perceived and actual adherence levels (e.g., high physician-perceived adherence and high claims-based adherence).

### Data analysis

Baseline patient and physician characteristics were compared using *χ*^2^ or Fisher’s exact test for categorical variables and Wilcoxon rank sum or *t*-test for continuous data. Atypical antipsychotic use patterns were stratified according to index medication and the groups were compared using *χ*^2^, Wilcoxon rank sum test or Kruskal–Wallis test. Data obtained from the physician surveys were grouped according to patient diagnosis, patterns of non-adherence and patient functionality. The primary endpoint was the concordance between physician responses, as indicated by decile category, and patient adherence data, also categorised by decile. A kappa coefficient (κ) was used to examine the agreement between the two adherence measures.

Power calculations were made using a worst case scenario for testing one adherence value against a second value. Assuming a 15% difference between the two adherence values with 80% power and *α* = 0.05 required for significance, the worst case estimate of the first adherence value of 0.50 was selected and tested against a value of 0.65 for the second adherence value because any different starting value, other than 0.50, would require fewer pairs (25.) Using these criteria, a sample size of at least 85 physician-patient pairs was determined to have 80% power to detect a difference of 15% in adherence at *α* = 0.05. Statistical analyses were performed using SAS version 9.1 (SAS Institute, Inc., Cary, NC, USA)

## Results

### Physician sample size

The physician sampling design was a stratified sample utilising physician contact information as well as physician panel size. Physicians treating patients with schizophrenia were determined first, with physicians treating bipolar patients comprising the rest of the sample. The targeted number of physician-patient surveys was 200.

A total of 5335 physicians were identified from the administrative claims of their patients. Of the 5335 identified physicians, invitations were sent to 3398 physicians for whom a fax number or mailing address was obtained and verified by the physicians’ office. Of the 3398 physicians receiving an invitation, 153 completed the survey for 214 patients, 45 were screened but never started the survey, 61 started but did not finish the survey and the remaining 3139 did not respond to the invitation. The response rate at the time the survey was closed because the targeted number of physician-patient surveys was reached was approximately 5%.

### Demographics

A total of 153 physicians completed the physician survey for 214 patients. Of this group of physicians, 92 (60%) completed surveys for one patient and 61 (40%) physicians completed surveys for two patients. The physician specialties were psychiatrists, 98 (64%); primary care physicians, including internists and general and family practice physicians, 34 (22%); and other specialties, 21 (14%). All patients with schizophrenia were treated by psychiatrists; the remaining types of physicians treated only patients with bipolar disorder. A higher percentage of physicians was located in the Midwest (64 physicians; 42%) compared with the West (36 physicians; 24%), South (29 physicians; 19%) or Northeast (24 physicians; 16%) regions of the US. Most physicians were men (98 physicians; 64%) and in group (72 physicians; 47%) or solo (55 physicians; 36%) practice. Twenty physicians (13%) practised in clinics and five (3%) practised in hospital settings, with one (1%) physician practicing in an unspecified type of setting. The majority of physicians had no academic affiliation (112 physicians; 73%) and no formal training in adherence (92 physicians; 60%). Of those with training, the top three types of training mentioned were during residency or fellowship, through courses presented at conferences and continuing medical education courses.

Descriptive characteristics of the 214 patients included in the administrative claims analysis are summarised in Table 1. Of the 214 patients, 162 (76%) had claims only with ICD-9-CM codes for bipolar disorder, 44 (21%) had claims only with ICD-9-CM codes for schizophrenia and 8 (4%) had claims with ICD-9-CM codes for bipolar disorder and schizophrenia.

**Table 1:** Patient characteristics*

		Medical claims with ICD-9-CM diagnosis codes		
		
Characteristic	Total patients (*N *= 214)	Schizophrenia only (*n *= 44)	Bipolar disorder only (*n *= 162)	Schizophrenia and bipolar disorder (*n *= 8)
**Gender, *n* (%)**				
Male	74 (34.58)	24 (54.55)	48 (29.63)	2 (25.00)
Female	140 (65.42)	20 (45.45)	114 (70.37)	6 (75.00)
Age, mean (±SD, median)	43.02 (±12.84, 45)	46.30 (±10.98, 46.50)	41.99 (±13.34, 45)	46.00 (±8.67, 44)
**Mental health conditions, *n* (%)**				
Depression	76 (35.51)	5 (11.36)	69 (42.59)	2 (25.00)
Anxiety disorder	49 (22.90)	3 (6.82)	45 (27.78)	1 (12.50)
Insomnia	21 (9.81)	2 (4.55)	18 (11.11)	1 (12.50)
Substance abuse				0
Alcohol	8 (3.74)	1 (2.27)	7 (4.32)	0
Opiates	4 (1.87)	0	4 (2.47)	0

* Variables identified from administrative claims data.

### Claims-based adherence

During the outcome study period, 206 patients (96%) received prescriptions for the index second-generation antipsychotic (Table 2). Eight (4%) patients had no prescription fills during that period and the MPR for these patients was set to 0. The mean MPR (±SD) during the 12-month presurvey period was 0.65 (±0.29) for patients with schizophrenia, 0.56 (±0.32) for those with bipolar disorder and 0.57 (±0.30) for those with both schizophrenia and bipolar disorder, indicating low-to-moderate adherence levels. By diagnosis, 17 patients (39%) with schizophrenia and 92 patients (57%) with bipolar disorder had low-to-moderate claims-based adherence levels (MPRs ≤70%), while 27 patients (61%) with schizophrenia and 70 patients (43%) with bipolar disorder had high adherence levels (MPRs >70%). Of the eight patients with medical claims for bipolar disorder and schizophrenia, five patients (62%) had low-to-moderate claims-based adherence levels and three patients (38%) had high medication adherence levels.

**Table 2:** Atypical antipsychotic treatment patterns during the 12 months prior to the physician survey*

		Medical claims with ICD-9-CM diagnosis codes		
		
Characteristic	Total patients (*N *= 214)	Schizophrenia only (*n *= 44)	Bipolar disorder only (*n *= 162)	Schizophrenia and bipolar disorder (*n *= 8)
Patients using index therapy, *n* (%)	206† (96.26)	43 (97.73)	155 (95.68)	8 (100)
**Fills of index therapy**‡,**mean (±SD, median)**				
Risperidone	10.50 (±4.41, 10.50)	10.89 (±2.98, 11.00)	10.23 (±5.28, 9.00)	n/a
Quetiapine	8.03 (±4.80, 8.50)	6.70 (±4.40, 6.50)	8.08 (±4.37, 9.00)	11.67 (±11.59, 10.00)
Olanzapine	7.63 (±3.69, 7.00)	8.42 (±4.06, 10.00)	7.32 (±3.51, 7.00)	6.75 (±4.03, 7.00)
Aripiprazole	7.15 (±4.56, 8.00)	9.50 (±3.89, 11.50)	6.79 (±4.58, 7.00)	n/a
Ziprasidone	6.86 (±4.17, 6.00)	8.50 (±3.87, 9.50)	6.33 (±4.53, 4.00)	5.00 (n/a, 5.00)
Paliperidone	4.75 (±3.85, 3.50)	2.50 (±0.71, 2.50)	5.50 (±4.23, 4.50)	n/a
Medication possession rate of index therapy, mean (±SD, median)	57.96 (±30.95, 66.03)	65.26 (±28.52, 74.77)	56.02 (±31.50, 63.42)	57.26 (±30.03, 54.28)

* Variables identified from administrative claims data.

† Eight patients had no atypical antipsychotic prescription filled during the 12 months prior to the physician survey and were excluded from this table.

‡ Five patients had two index atypical antipsychotic medications.

### Physician-patient concordance

Physicians generally overestimated the adherence levels of their patients. Of 214 patients, physicians perceived 163 (76%) of their patients to be adherent to medication at least 71% of the time during the past 12 months, compared with 100 (47%) patients who had a MPR value that was greater than 70% for the same 12 month period (*κ* = 0.0572; p = 0.1908). Of the 214 paired patient-physician assessments of adherence, physician estimates were concordant with claims-based adherence levels for 99 patients (46%), physicians overestimated patient adherence for 94 patients (44%) and physicians underestimated patient adherence for 21 patients (10%) (Table 3). Although, 114 patients had low-to-moderate claims-based adherence (0–70%), physicians categorised 82 (72%) of those patients as having high adherence levels (71–100%) (Table 3), and only 32 (28%) as having low-to-moderate adherence (0–70%). Of the 100 patients with high claims-based adherence levels (70–100%), physicians perceived 19 (19%) to be moderately adherent (31–70%), while the remaining 81 (81%) were believed to be highly adherent (71–100%).

**Table 3:** Claims-based adherence and physician-reported adherence

	Physician-reported adherence														
	
	Total patients (*N *= 214)					Schizophrenia diagnosis codes only (*n *= 44)					Bipolar disorder diagnosis codes only (*n *= 162)				
			
	Adherence					Adherence					Adherence				
									
Claims-based	Low (0–30%)	Moderate (31–70%)	High (71–100%)	Kappa coeffi-cient	p value	Low (0–30%)	Moderate (31–70%)	High (71–100%)	Kappa coeffi-cient	p value	Low (0–30%)	Moderate (31–70%)	High (71–100%)	Kappa coeffi-cient	p value
Low adherence (0–30%)	5	12	32	0.0572	.1908	0	0	7	0.0088	.9089	4	12	24	0.0478	.3522
Moderate adherence (31–70%)	2	13	50			0	1	9			2	12	38		
High adherence (71–100%)	0	19	81			0	2	25			0	17	53		

Kappa coefficient assesses the degree of agreement between physician-reported adherence and claims-based adherence; a large Kappa indicates a strong level of agreement. The Kappa test is used to test the null hypothesis of no agreement. The small value of Kappa and p value indicate no agreement between physician-reported adherence and claims-based adherence. There are eight patients with both schizophrenia and bipolar disorder diagnosis codes that are included only in the total patients’ adherence part of the above table.

Physician adherence perceptions were in concordance with claims-based estimates for 26 of the 44 patients (59%) with schizophrenia and 69 of the 162 patients (43%) with bipolar disorder. Among patients with schizophrenia, 16 (94%) of the 17 patients with low-to-moderate claims-based adherence (0–70%) were perceived by physicians to have high adherence (71–100%), whereas 2 (7%) of 27 patients with high claims-based adherence (71–100%) were perceived by physicians to have low-to-moderate adherence (0–70%). Among the 162 patients with bipolar disorder, physicians overestimated adherence for 62 (67%) of 92 patients with low-to-moderate claims-based adherence (0–70%) and underestimated adherence in 17 (24%) of 70 patients with high claims-based adherence (71–100%) (Table 3).

Physician characteristics were compared with concordance levels. Of the 153 responding physicians, 50 (33%) perceived patient adherence to be greater than the claims-based adherence, 11 (7%) perceived patient adherence to be less than the claims-based adherence, 57 (37%) were concordant with claims-based adherence levels and 35 (23%) physicians, each responding for two patients, had mixed adherence classifications (e.g., one patient was concordant and the other was overestimated). Formal training on medication adherence appeared to have no effect on the physicians’ ability to correctly estimate adherence levels in their patients. Of the 57 physicians with concordant estimates, 40 (70%) had no formal adherence training, although more physicians in both the underestimated (6 of 11 physicians, 55%) and overestimated (28 of 50 physicians, 56%) concordance groups also lacked formal training. No statistically significant differences were found between the concordance groups with regard to gender, academic affiliation, type of practice setting or geographical region. Neither the number of total patients in the practice nor the number of patients with schizophrenia or bipolar disorder affected the ability of physicians to predict medication adherence.

All the physicians indicated that they discussed the importance of medication adherence with their patients, with 97% noting that they ‘always’ (104 physicians; 68%) or ‘often’ (44 physicians; 29%) discussed adherence. All the physicians also answered that they ‘always’ asked their patients about adherence to the prescribed treatment regimen and about problems with their medication. Most physicians (150; 98%) stated that they discussed the importance of routine follow-up appointments with patients.

## Discussion

This study compared physicians’ perceptions of patients’ adherence to oral second-generation antipsychotics with the patients’ actual medication usage as determined from pharmacy claims. Our approach was different from those of other studies assessing physician perceptions of adherence. Previous studies assessing adherence to oral antipsychotics have compared physician estimates of adherence with patient self-reports, pill counts, electronic monitoring, electronic refill information and blood monitoring (22, 23, 25, 26). In a review of antipsychotic adherence research, the most commonly used method for assessing adherence was patient self-report (25). Electronic refill records were used in only a small percentage of studies (8 of 161) and pharmacy claims were not used in any study (25). In studies where physicians estimated adherence for individual patients, the assessment period was short, the patient population was small and drawn from a convenience sample, and it was unclear whether the physicians and patients had an established long-term relationship (22, 23). In contrast, our study asked physicians to estimate adherence of specific patients in their practice whom they had seen regularly for at least 1 year. The hypothesis was that familiarity with each patient’s history and behaviours would enable physicians to make more informed assessments of treatment adherence. In addition, research in patients receiving second-generation antipsychotics revealed that patients who had previous antipsychotic prescriptions were more likely to follow their medication regimen for a longer period than those receiving antipsychotics for the first time (27). Together, these elements—experienced physicians who have an established relationship with patients who are accustomed to their antipsychotic regimen—represent a best-case scenario and promise a high degree of accuracy in physicians’ ability to estimate individual adherence rates.

Our results, however, support previous findings of physician overestimation of adherence (4, 18, 19, 21, 28). Despite having treated the patients for at least 1 year, the surveyed physicians indicated approximately three-quarters of their patients were highly adherent to their therapeutic regimen, while claims-based data showed that fewer than half of the patients were highly adherent. The physicians also classified as highly adherent 82 (72%) of 114 patients who were found to have low-to-moderate claims-based adherence. Neither formal training in adherence nor the experience level of the physicians improved the physicians’ ability to estimate performance.

Discrepancies between physician perceptions of adherence and patient perspectives have been studied in many chronic illnesses, including mental health conditions (4, 6, 18–21, 28–31). Our results provide further support for the established finding that physicians overestimate adherence, whether the physicians’ estimates are based on higher level, general assessments of patients with a particular disease state, a moderate level of specificity, such as the patients in a general practice population, or, as our study found, at the individual patient level (4, 18–21, 28). Familiarity with the patient did not improve the ability of the physicians to estimate treatment adherence. Previous research on physician estimates of patient adherence, whether in mental health or other therapeutic areas, was directed to higher levels of assessment and, to our knowledge, our study is novel in comparing physician estimates of adherence of individual patients with claims data. A similar design was used by Copher et al. in their study of adherence among patients with osteoporosis; however, although physicians were surveyed about patients in their own practises, the assessments were kept at the practice level rather than the patient level (28).

To improve adherence, researchers have called for treatment teams to try to better understand the reasons patients fail to take medications (32). That strategy presupposes the ability of physicians to recognise non-adherence in their patients. Our results show that physicians have difficulty estimating adherence, even in patients they see regularly.

The study had several limitations. Patients and their physicians were identified from a large administrative claims database with claims from employer-sponsored health plans in the US. The results from this study may not be generalisable to other physician and patient populations because of the relatively small number of physicians responding to the survey and the patients about whom they were asked. The physicians who took part in this study were asked about high functioning patients that were covered by an employer’s health plan and their responses may not be generalisable to other populations including patients with public health insurance or no insurance. Likewise, the patients in the study population may not be generalisable to other patients with mental disorders, or to patients in countries other than the US with different systems of health care. The majority of patients with schizophrenia in the US do not have commercial health insurance; usually they are covered by public health insurance or they are uninsured (33). The patients with schizophrenia included in this study were covered by their own employers’ health plans or the plans of their spouses. This suggests these patients were functioning at a level that enabled them to maintain a relationship or steady employment.

Administrative claims data may contain diagnostic or treatment coding errors, and although there was a record of prescriptions filled, there was no way to determine whether the patients actually took the medication as prescribed and likely represents an underestimation of adherence.The patients also could have received medication samples or had prescriptions filled by pharmacies outside the health plans captured in the HIRD. Although many antipsychotics have FDA indications for bipolar disorder, they are not necessarily recommended for or prescribed to all individuals with bipolar disorder. When evaluating antipsychotic adherence using the MPR in individuals with bipolar disorder, we could not distinguish between gaps in treatment that were because of non-adherence vs. gaps in treatment or complete discontinuation that were clinically indicated. Finally, physicians were asked to estimate adherence over a 12-month period which may be subject to recall bias whereas the claims-based estimates of adherence were based on prescription fill data.

## Conclusions

These results support previous findings showing discordance between physician perceptions of patient adherence and claims-based adherence measured through analysis of pharmacy claims. By merging physician survey data with patient pharmacy claims data, the current study assessed the ability of prescribing physicians to estimate the adherence of specific patients with schizophrenia or bipolar disorder in their practices and compared those estimates with actual prescription fill behaviours. However, care must be taken because these results for commercially insured patients with schizophrenia or bipolar disorder may not be generalised to other populations.
